# Current Status of Barriers to mHealth Access Among Patients With Stroke and Steps Toward the Digital Health Era: Systematic Review

**DOI:** 10.2196/54511

**Published:** 2024-08-22

**Authors:** Atsadaporn Niyomyart, Suebsarn Ruksakulpiwat, Chitchanok Benjasirisan, Lalipat Phianhasin, Kabtamu Nigussie, Sutthinee Thorngthip, Gazi Shamita, Jai Thampakkul, Lidya Begashaw

**Affiliations:** 1 Ramathibodi School of Nursing, Faculty of Medicine Ramathibodi Hospital, Mahidol University Bangkok Thailand; 2 Department of Medical Nursing, Faculty of Nursing, Mahidol University Bangkok Thailand; 3 Department of Psychiatry, College of Health and Medical Sciences, Haramaya University Harar Ethiopia; 4 Department of Nursing Siriraj Hospital, Faculty of Medicine Siriraj Hospital, Mahidol University Bangkok Thailand; 5 Department of Dermatology, School of Medicine, Case Western Reserve University Cleveland, OH United States; 6 Case School of Engineering, Case Western Reserve University Cleveland, OH United States; 7 Jack, Joseph and Morton Mandel School of Applied Social Sciences, Case Western Reserve University Cleveland, OH United States

**Keywords:** digital health, mHealth, barrier, stroke, systematic review, mobile phones

## Abstract

**Background:**

Mobile health (mHealth) offers significant benefits for patients with stroke, facilitating remote monitoring and personalized health care solutions beyond traditional settings. However, there is a dearth of comprehensive data, particularly qualitative insights, on the barriers to mHealth access. Understanding these barriers is crucial for devising strategies to enhance mHealth use among patients with stroke.

**Objective:**

This study aims to examine the recent literature focusing on barriers to mHealth access among patients with stroke.

**Methods:**

A systematic search of PubMed, MEDLINE, Web of Science, and CINAHL Plus Full Text was conducted for literature published between 2017 and 2023. Abstracts and full texts were independently screened based on predetermined inclusion and exclusion criteria. Data synthesis was performed using the convergent integrated analysis framework recommended by the Joanna Briggs Institute.

**Results:**

A total of 12 studies met the inclusion criteria. The majority were qualitative studies (about 42%), followed by mixed methods (25%), pilot studies (about 17%), nonrandomized controlled trials (about 8%), and observational studies (about 8%). Participants included patients with stroke, caregivers, and various health care professionals. The most common mHealth practices were home-based telerehabilitation (30%) and poststroke mHealth and telecare services (20%). Identified barriers were categorized into two primary themes: (1) at the patient level and (2) at the health provider-patient-device interaction level. The first theme includes 2 subthemes: health-related issues and patient acceptability. The second theme encompassed 3 subthemes: infrastructure challenges (including software, networking, and hardware), support system deficiencies, and time constraints.

**Conclusions:**

This systematic review underscores significant barriers to mHealth adoption among patients with stroke. Addressing these barriers in future research is imperative to ensure that mHealth solutions effectively meet patients’ needs.

## Introduction

### Background

Stroke, a leading cause of disability and mortality worldwide, necessitates immediate and ongoing interventions for optimal recovery [1]. While mobile health (mHealth) technologies offer promising solutions for chronic disease management, understanding and addressing the unique barriers faced by patients with stroke is crucial for ensuring their equitable access and optimal use [2,3]. While previous research has explored mHealth adoption in various populations, the cognitive impairments [4], rehabilitation needs [5], and potential technology literacy limitations of patients with stroke [6] require distinct consideration. mHealth, encompassing the use of mobile devices, applications, and wireless communication devices, offers promising avenues to deliver personalized health care solutions to patients with stroke beyond the confines of traditional health care settings [3,7].

The health care landscape has evolved with the proliferation of mobile devices and the availability of high-speed internet connectivity. Consequently, mHealth platforms have emerged as tools that can potentially bridge the gap between health care providers and patients, enabling continuous monitoring, real-time communication, and targeted interventions [8,9]. In the context of stroke, where timely interventions and ongoing support are crucial, mHealth can revolutionize poststroke care by providing patients with access to rehabilitation exercises, medication reminders, educational resources, and even telemedicine consultations [3,6,10]. Despite the promise of mHealth, realizing its benefits for patients with stroke is contingent upon understanding and mitigating the barriers that hinder its widespread adoption and use. Previous research has underscored the importance of identifying these barriers to ensure equitable access and optimal use of mHealth services among patients with stroke [11]. While existing literature has explored mHealth adoption in various populations, the unique challenges faced by patients with stroke deserve particular attention due to the nature of their condition, potential cognitive impairments, and the necessity of tailored interventions. The period between 2017 and 2023 has witnessed substantial advancements in mHealth technologies, health care policies, and the prevalence of mobile device usage among diverse demographic groups. Consequently, there is a need to assess the current status of mHealth access among patients recovering from stroke within this evolving landscape. By systematically reviewing the literature and synthesizing recent findings, this study aims to delineate the barriers that impede access to mHealth services among patients with stroke, analyze their impact, and propose actionable recommendations for stakeholders in the health care ecosystem.

In light of the growing importance of digital health and its potential benefits for patients with stroke, investigating the barriers to mHealth access is a critical step toward ensuring that these advancements are inclusive and patient centered. By identifying these barriers and proposing strategies to overcome them, this study seeks to contribute to the advancement of stroke care in the digital health era.

### Objective

This study seeks to evaluate recent literature, focusing on the notable barriers to accessing mHealth services within the population of patients with stroke. Through this assessment, the study aims to offer recommendations aimed at effectively addressing these barriers and propelling progress in this critical domain of health care.

## Methods

### Identify Relevant Studies

We adhered to the PRISMA (Preferred Reporting Items for Systematic Reviews and Meta-Analyses) guidelines [12] while conducting this systematic review. This approach guided the presentation of the flow diagram depicting the identification, screening, exclusion, and inclusion of literature. To identify studies published between 2017 and 2023 that reported barriers to mHealth access among the patients with stroke, we systematically searched 4 electronic databases: PubMed, MEDLINE, Web of Science, and CINAHL Plus Full Text, on September 1, 2023. Our search involved the combination of terms: (Stroke* or Cerebrovascular Accident* or CVA* or Cerebrovascular Apoplexy or Brain Vascular Accident* or Cerebrovascular Stroke* or Apoplexy or Cerebral Stroke* or Acute Stroke* or Acute Cerebrovascular Accident* or Brain Infarction* or Brain Infarct* or Anterior Circulation Brain Infarction or Brain Venous Infarction* or Venous Brain Infarction* or Anterior Cerebral Circulation Infarction or Posterior Circulation Brain Infarction or Cerebral Infarction* or Cerebral Infarct* or Left Hemisphere, Infarction, Cerebral or Subcortical Infarction* or Posterior Choroidal Artery Infarction or Anterior Choroidal Artery Infarction or Hemorrhagic Stroke* or Subarachnoid Hemorrhagic Stroke* or Intracerebral Hemorrhagic Stroke* or Ischemic Stroke* or Ischaemic Strok* or Cryptogenic Ischemic Stroke* or Cryptogenic Stroke* or Cryptogenic Embolism Stroke* or Wake-up Stroke* or Acute Ischemic Stroke* or Embolic Stroke* or Cardioembolic Stroke* or Cardio-embolic Stroke* or Thrombotic Stroke* or Acute Thrombotic Stroke*) AND (Tele-Referral* or Virtual Medicine or Tele Intensive Care or Tele ICU or Mobile Health or mHealth or Telehealth or eHealth or Remote Consultation or Teleconsultation* or Telenursing or Telepathology or Teleradiology or Telerehabilitation* or Remote Rehabilitation* or Virtual Rehabilitation*) AND (Barrier*) using Boolean operators. Additionally, we manually searched the reference lists of the included studies to ensure inclusivity. All identified references were cataloged using EndNote.

### Study Selection

We conducted a 3-step selection process. First, we screened titles and abstracts to identify eligible studies. Then, we assessed the full text to determine relevance. Finally, inclusion criteria were applied to ensure that only studies aligned with our objectives were included. Conversely, exclusion criteria were used to eliminate literature not pertinent to the review (see Textbox 1).

**Inclusion and exclusion criteria.**

**Inclusion criteria:**

- The study included patients with stroke aged 18 years or older. The study may also encompass other populations, such as caregivers and health care teams, but patients with stroke must be included
- Original studies using quantitative, qualitative, or mixed methods approaches
- Studies that identified barriers to accessing mobile health (mHealth) services among patients with stroke (all types of strokes are eligible)
- In this study, mHealth is identified as the practice of medicine and public health supported by mobile devices. It encompasses the use of mobile devices, such as smartphones, tablet computers, personal digital assistants, and others, for health services, information, and data collection [13]
- Studies conducted in various settings, including inpatient, outpatient, or home environments
- Studies published in the English language
- Studies published between January 2017 and December 2023

**Exclusion criteria:**

- Studies that did not involve patients were excluded.
- Exclusion of conference proceedings, abstracts, review articles, theoretical papers, protocols, dissertations, letters to the editor, opinion (viewpoint) pieces, statement papers, government documents, or working papers

### Data Extraction

For this review, we used a standardized data extraction chart that included the following data points for each study: Reference, country, year, study design, total sample size, target population, participant age (years), main study aim, main findings, presence of mHealth in included studies, key barriers to accessing mHealth, and further research implications.

### Data Synthesis

For the data synthesis of the included studies in this review, we used the convergent integrated analysis framework recommended by the Joanna Briggs Institute (JBI) for systematic reviews. In this process, themes will be derived from the key findings of the included studies by analyzing both commonalities and distinctions among the primary findings related to key barriers in accessing mHealth. Furthermore, subthemes will be extracted as necessary, aligning with the specific focus of the corresponding findings, akin to the methodology used by qualitative researchers [14].

## Results

### Search Results

**Figure 1:**
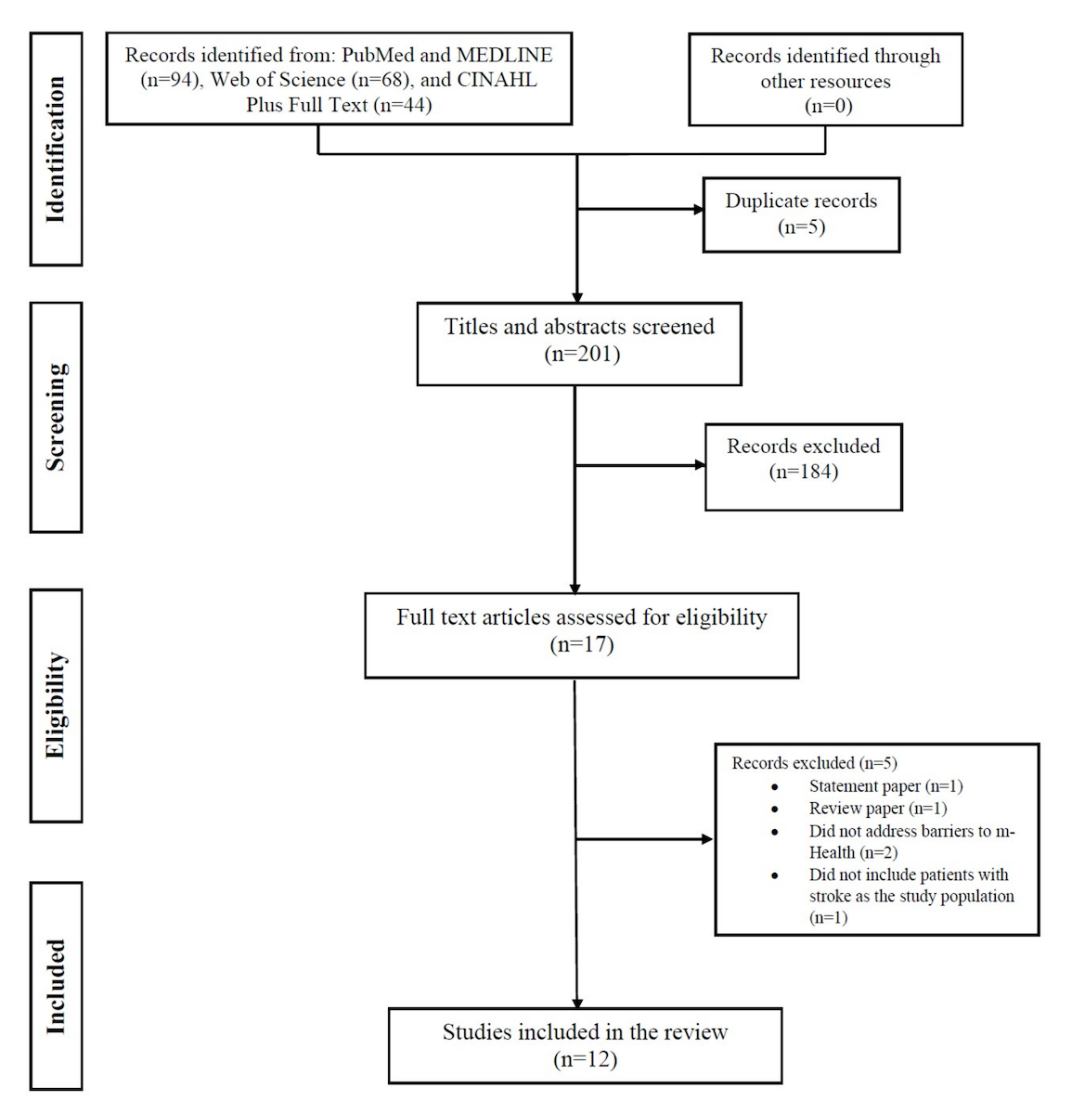
Flowchart diagram displaying the selection method of qualified studies.

Following the PRISMA guidelines [12], we initially identified a total of 206 articles. Out of these, 94 were obtained from PubMed and Medline, 68 from Web of Science, and 44 from CINAHL Plus Full Text. No additional articles were found from other sources. After a thorough review, we identified and removed 5 duplicate articles. Subsequently, the remaining articles underwent screening based on their titles and abstracts, following the inclusion and exclusion criteria (Textbox 1). At this stage, 184 articles did not meet the inclusion criteria and were excluded, leaving us with 17 articles eligible for full-text screening. During the full-text screening phase, 5 articles were excluded. Of these, 1 was identified as a statement paper, 1 was a review paper, 2 did not address barriers to mHealth, and 1 did not include patients with stroke as the study population. Consequently, 12 studies were included in the review (Figure 1).

### Data Summary

Multimedia Appendix 1 [15-26] provides a summary of each included study, including reference, country, year, study design, total sample size, target population, participant age (years), main study aim, main findings, presence of mHealth in included studies (optional), key barriers to accessing mHealth, and implications for further research.

### Description of Included Studies

Table 1 shows that all included studies were published between 2017 and 2023, with the most publications in 2023 (n=5, 42%) and 2022 (n=3, 25%). Among these, one publication was found each in 2017, 2018, 2020, and 2021. Most of the included studies were conducted in the United States (n = 4, 33%). Other countries include the United Kingdom (n=2, 17%), Hong Kong (China) (n=2, 17%), Brazil (n=1, 8%), and the Netherlands (n=1, 8%). In terms of study design, qualitative studies were the most popular (n=5, 42%), followed by mixed methods (n=3, 25%), pilot studies (n=2, 17%), nonrandomized control trials (n=1, 8%), and observational studies (n=1, 8%). Among the patients with stroke included in the study, there was no specific stroke type identified (n=9, 64%), ischemic stroke (n=2, 14%), hemorrhagic stroke (n=2, 14%), and chronic hemiplegic stroke (n=1, 7%).

**Table 1:** The characteristics of the included studies.

Characteristics				Values, n^a^ (%)
**Publication year**				
	2023		5 (42)	
	2022		3 (25)	
	2021		1 (8)	
	2020		1 (8)	
	2018		1 (8)	
	2017		1 (8)	
**Country**				
	United States		4 (33)	
	United Kingdom		2 (17)	
	Hong Kong (China)		2 (17)	
	Brazil		1 (8)	
	The Netherlands		1 (8)	
	Singapore		1 (8)	
	Republic of Korea		1 (8)	
**Study design**				
	Qualitative study		5 (42)	
	Mixed methods		3 (25)	
	Pilot study		2 (17)	
	Nonrandomized controlled trial		1 (8)	
	Observational study		1 (8)	
**Target population**				
	**Patients with stroke**			
		Not specify stroke type	9 (64)	
		Ischemic stroke	2 (14)	
		Hemorrhagic stroke	2 (14)	
		Chronic hemiplegic stroke	1 (7)	
	**Health care professionals and other**			
		Caregiver	3 (14)	
		Clinician	1 (14)	
		Rehabilitation therapist	1 (14)	
		Occupational therapist	1 (14)	
		Nurse	1 (14)	
**Total sample size**				
	1-50		9 (75)	
	>50-100		1 (8)	
	>100-200		1 (8)	
	>200		1 (8)	
**mHealth^b^ in included studies**				
	A home-based telerehabilitation [15-17]		3 (30)	
	A poststroke mHealth and telecare service [18,19]		2 (20)	
	The remote physical exercise program [20]		1 (10)	
	A home-based web-based clinic [21]		1 (10)	
	Self-administered VR^c^ telerehabilitation [22]		1 (10)	
	The Homecare Arm Rehabilitation System (MERLIN) [23]		1 (10)	
	Remote CCT^d^ and metacognitive strategy (MST^e^) training [24]		1 (10)	

^a^The number of included studies in which one study may include more than one characteristic (eg, target population, mobile health, etc).

^b^mHealth: mobile health.

^c^VR: virtual reality.

^d^CCT: computerized cognitive.

^e^MST: metacognitive strategy.

Additionally, caregivers (n=3, 43%), clinicians (n=1, 14%), rehabilitation therapists (n=1, 14%), occupational therapists (n=1, 14%), and nurses (n=1, 14%) were included in the study. Sample sizes ranged from 1 to 50 (n=9, 75%), over 50 to 100, over 100 to 200, and over 200 (all n=1, 8%). The most common mHealth practices in the included studies were home-based telerehabilitation (n=3, 30%) and poststroke mHealth and telecare service (n=2, 20%). The rest of mHealth consisted of remote physical exercise, a home-based web-based clinic, self-administered virtual reality (VR) telerehabilitation, and home care arm rehabilitation, as well as remote cognitive (CCT) and metacognitive strategy training (MST; all n=1, 10%).

### Description of Barriers to mHealth Access in Patients With Stroke

Multimedia Appendix 1 [15-26] provides a summary of the barriers to mHealth accessibility experienced by patients with stroke. Having used the convergent integrated analysis framework recommended by JBI for systematic reviews [14], we can discern two primary themes: (1) at the patient-level and (2) at the health provider-patient-device interaction level. The first theme included 2 subthemes: health-related barriers and patient acceptability. The second theme encompassed 3 subthemes: infrastructure (inclusive of software, networking, and hardware), deficiencies in support systems, and constraints on available time (Table 2).

**Table 2:** Barriers to mobile health (mHealth) access.

Study	Barriers to mHealth Themes						
	The patient-level		The health provider-patient-device interaction level				
	Health-related barriers	Patient acceptability	Infrastructure			Deficiencies in support systems	Constraints on available time
			Software	Networking	Hardware		
Dodakian et al [15]	✓				✓		✓
Tyagi et al [26]			✓	✓	✓		
Dunne et al [25]		✓					✓
Torriani-Pasin et al [20]	✓					✓	✓
Lam et al [21]				✓			
Morse et al [22]		✓		✓	✓	✓	
Spits et al [23]		✓				✓	
Bhattacharjya et al [16]			✓			✓	✓
Chung et al [17]		✓					
Jaywant et al [24]		✓		✓	✓	✓	
Ramaswamy et al [18]			✓		✓		
Wong et al [19]			✓			✓	
Number of included studies, n (%)	2 (17)	5 (42)	4 (33)	4 (33)	5 (42)	6 (50)	4 (33)

### The Patient Level

#### Health-Related Barriers

Our results indicate that health-related issues are among the barriers to mHealth access for patients with stroke. A study evaluating a home-based telerehabilitation system in patients with chronic hemiparetic stroke found that fatigue due to illness prevented patients from using the Home-Based Telerehabilitation Program [15]. Similarly, a clinical trial study from Brazil aimed at determining adherence and barriers to attending a remote physical exercise program for individuals after stroke shows that health-related factors such as a lack of motor skills, physical fitness, exercise-related pain, and other constraints are significant barriers preventing them from using the remote physical exercise program [20]. For instance, in a qualitative study conducted with survivors of stroke experiencing partial visual loss, caregivers and occupational therapists revealed that patients believed their lack of confidence and fear of using technology prevented them from learning new things, and their readiness to embrace technology was a barrier to telerehabilitation [25].

#### Patient Acceptability

Five studies in our review have highlighted patient acceptability as the primary barrier to mHealth [17,22-25]. For example, a mixed-method study identified facilitators and barriers to using self-administered VR telerehabilitation, suggesting that a lack of experience or confidence with technology hindered access to self-administered VR telerehabilitation [22]. Moreover, a pilot study found that patients with stroke, especially older adults with stroke, may have limited digital literacy skills, making it challenging to use mHealth tools effectively. Additionally, a study points out that participants' attitudes and acceptance of mHealth interventions may vary; some individuals may be hesitant or skeptical about using technology for health care purposes [24].

### The Health Provider-Patient-Device Interaction Level

#### Infrastructure

##### Software

The complexity of mHealth software can pose challenges for patients in its usage [16,18,19,26]. For instance, a qualitative study conducted in Singapore explored the perceived barriers and facilitators of telerehabilitation by patients with stroke, caregivers, and rehabilitation therapists. The study noted that teletherapists encountered difficulties conducting comprehensive patient assessments remotely [26]. Similarly, patients perceived limitations in the variety and scope of rehabilitation exercises available through telerehabilitation, attributing this to issues related to the interface and design of the remote platform [26]. In line with this, another study aimed to explore potential mHealth apps to aid survivors of stroke with poststroke care and determine how demographic variables affect app preferences. This study found that the complexity of app usage acted as a barrier, preventing some users from using these apps effectively [18]. Considering apps on outdated smartphones or operating systems can result in challenges for patients when running and installing complex apps. In addition, there is a noticeable mismatch between the technical complexity of the apps and the users’ capacities, as they lack the knowledge to address issues like nonfunctionality, showdowns, freezing, and crashes in the apps [27,28].

##### Network

In our review, 4 included studies highlighted the significance of network issues as a critical barrier to mHealth access [21,22,24,26]. Of these, 2 studies emphasized the importance of reliable internet access for effective remote interventions. Poor internet connectivity or limited access to high-speed internet in certain areas can impede the successful implementation of mHealth interventions [24,26]. Furthermore, the high cost and limited availability of internet access to support the equipment in survivors of stroke’s homes can prevent them from accessing mHealth services [21,22]. Patients and health care professionals mainly rely on an internet-based system where health care providers must promptly update patient information. On the other hand, patients can use the apps to receive health updates and discuss their symptoms with health care providers effectively [29,30]. Thus, the absence of a digital option can restrict internet access and cause challenges, both at the patient’s house and within the health care facility. However, adopting an offline mode can make it difficult to transfer data, which typically relies on web-based connectivity.

##### Hardware

Hardware malfunctions, such as those in mobile phones or computers, are considered significant factors affecting the delivery of mHealth services [15,18,22,24,26]. For instance, in a pilot study aiming to implement a home-based Telerehabilitation Program that included arm motor therapy games, therapeutic arm exercises, remote stroke education, and videoconferencing, hardware malfunctions limited patients’ access to the intervention [15]. Moreover, a qualitative study highlighted equipment setup–related difficulties as a barrier to mHealth access, with patients with stroke encountering challenges in setting up and using the required equipment [26]. For example, getting ready for video-recorded exercise can be a challenge among patients with stroke when setting up the iPad, sensors, and monitoring equipment for heart and blood pressure [31]. In addition, specific details, such as the correct connection of the limb sensor node to assess the patient’s range of motion and troubleshoot unexpected hardware problems, may not be straightforward, even though general instructions are provided. Another study focusing on remote interventions, which often rely on technology, noted that one potential barrier could be the accessibility of the necessary technology (eg, smartphones, computers, etc) for individuals with chronic stroke, especially those who may not be familiar with or have easy access to such devices [24].

#### Deficiencies in Support Systems

Lack of support systems is a significant barrier to mHealth access, as indicated by 6 included studies in our review [16,19,20,22-24]. For instance, a qualitative research study investigated the experiences of 13 survivors of stroke and health care providers regarding the use of a poststroke telecare service [19]. The study found that the lack of general guidelines for operating a telecare service, technical issues, and limited human resources presented challenges to patients in using the service (eg, difficulties in joining Zoom meetings and troubleshooting) [19]. Another study also emphasized the importance of adequate training and support for participants using mHealth tools. This study recommended initial in-person sessions to familiarize participants with the technology, indicating that additional support might be necessary for some individuals [24]. In a mixed methods study aimed at identifying facilitators and barriers to the use of self-administered VR telerehabilitation, it was mentioned that a lack of an exercise companion and a safe environment for exercising prevented some individuals from using self-administered VR telerehabilitation [20].

#### Constraints on Available Time

In our study, we have found that constraints on available time are considered a barrier to mHealth, as mentioned in 4 published studies [15,16,20,25]. For example, 1 included study stated that conflicts with other medical appointments prevent patients with stroke from completing a home-based telerehabilitation program [15]. Additionally, work commitments are also a factor preventing patients with from using mHealth at home [20]. This issue is not limited to patients alone; it also affects health care providers. Qualitative research revealed that time-related issues are prevalent among providers, such as occupational therapists who reported having limited time to spend with survivors of stroke when providing mHealth interventions [25]. Additionally, a prior study highlighted the importance of the design and development stages of mHealth in reducing disruptions to health care providers’ established workflows [32]. The study emphasized the transformation of functionalities into practical health tools, aiming to facilitate the integration of mHealth into their existing structured workflows, ultimately influencing increased engagement in the adoption of mHealth.

## Discussion

The study aimed to explore recent literature to uncover possible barriers to mHealth access among patients with stroke. Although mHealth offers an important option for accessing needed health services, and people with stroke are currently using it for various purposes, this study identified several barriers inhibiting its use. One such barrier is health-related issues. Prior studies have shown that 25%-85% of survivors of stroke report experiencing fatigue, regardless of the severity of their condition [33,34]. As fatigue develops over time, it can result in the inability to perform basic activities, such as dressing and eating, and eventually limit more complex activities like shopping and preparing meals [35]. Considering that the study included complex telerehabilitation activities (eg, games, therapeutic programs, education, and videoconferencing), access to the program among patients with stroke could be limited due to fatigue. It is also important to consider a patient's age when examining fatigue, whether they are young or old [36-38]; this finding aligns with our study, which included patients aged 40 and older who had experienced strokes.

Despite the rise of mHealth to connect patients to health services outside of a clinical setting, our study found that patients’ readiness and acceptance were barriers to accessing mHealth. Similar to previous studies, patients with stroke reported that visual and physical impairments hindered their access to mHealth, as they lacked the confidence to communicate electronically and were concerned about making mistakes during remote interactions [25,39]. These impairments, affecting sight and physical activity, may contribute to reduced readiness for mHealth. Additionally, digital health literacy should be considered, as patients with stroke, particularly those in the older population, may have difficulty obtaining, interpreting, and evaluating health information through digital sources [40]. A similar finding was observed in another study, where older patients with stroke had less access to phone or video telephone visits due to a lack of digital literacy and access, as well as a lack of experience using such technology [41]. In terms of acceptability, patients with stroke who perceived m-Health as useless (e.g., only making phone calls) restricted their access to mHealth applications, such as mobile home-based exercise programs [42].

One of the main obstacles for mHealth is the availability of adequate infrastructure to provide effective and comprehensive care to patients with stroke. The review identified the limitations of the current software in conducting patient assessments and delivering a wide range of rehabilitation exercises. Since the main goal of rehabilitation is to improve the patient’s physical impairments, a health care provider needs to perform physical exams to determine and evaluate the intervention. Without such contact through mHealth, the health care provider’s assessment ability is compromised, and the assessment accuracy might be reduced. However, previous studies have proposed remote methods to assess patients with stroke, such as internet-based telerobotic devices, videoconferencing, camera-based artificial intelligence (AI) models with wearable sensors, and remote Fugl-Meyer Assessment protocols [43-46]. The validity of the proposed methods was still low, and additional equipment and advanced software are required to capture the more accurate physical examination details.

The studies in the review also highlighted the narrow range of exercises the patients could perform through mHealth. A systematic review of home-based technologies for stroke rehabilitation revealed technology that increases the variety of exercise, such as games, telerehabilitation, robotic devices, VR devices, and tablets. This review also pointed out the limitations of each technology, which is consistent with our findings that the equipment requires proper guidance in setting up and using to achieve the therapeutic goals [47].

Besides having the proper software and hardware for mHealth services for patients with stroke, the studies also highlighted poor or limited internet connectivity as a major barrier to implementing mHealth. Many mHealth interventions require timely monitoring, access to the database, or health care. Some studies have explored alternative solutions to enhance mHealth delivery in areas with limited or nonexistent internet access. For example, some studies have used Firebase, a local offline database, or caching the user interface and assets to help patients stay connected to the information if the patients lose an internet connection [48-50]. Others have integrated communities or schools as a link between larger hospitals and patients who cannot access the internet [51,52]. Nevertheless, internet connectivity remains a critical obstacle for mHealth, as it was considered a super social determinant of health that affects other aspects of health equity [53]. Therefore, promoting affordable and reliable internet access would be beneficial, especially for those who face disadvantages in accessing health care.

Our review reported deficiencies in support systems in using mHealth, including the need for general guidelines in using the services, technical issues, additional support, the lack of an exercise companion, and a safe environment. The lack of general guidelines or instructions in using a relative innovation can influence patients’ or caregivers’ decision to use and continue use. Our findings are congruent with previous longitudinal studies of the continued use of mHealth apps, indicating that users will not be motivated to continue service if they do not understand how the system works based on their initial interactions [54]. Along with a limited support system, most of the participants in our studies are notably older adults, and a prior study revealed that older adults, in general, have a lower rate of adopting mHealth [55]. The adoption of mHealth is not a simple process and requires not only technical assistance but also human support to enhance their experience and interaction with mHealth [56,57]; thus, concise and clear guidelines are essential in facilitating and engaging their usage. Another study also points out that mobile health for older adult patients used an aging barrier framework to explore usability problems and reported that participants have difficulties understanding the navigation structure of the apps and overseeing important text, buttons, and icon elements [58]. Therefore, clear guidelines and instructions should be considered when developing mHealth for survivors of stroke.

Regarding technical issues, it can be solved or reduced by additional support. Our findings align with a study evaluating patients’ experience with the usability of a diabetes mHealth system. Technical issues related to functionality or operation can impact the overall usability of apps, such as challenges in deleting or inputting glucose values, resulting in difficulties in accurately interpreting value ranges (51). Furthermore, participants who encounter technical issues may struggle to navigate through different functions; for instance, participants have reported experiencing navigation difficulties while reporting glucose diary values. Prior studies have also highlighted the significance of app navigation for patients with stroke who face challenges in managing their medication [28,59]. Thus, navigation within apps is crucial as if it is difficult and user-unfriendly, it can impact patients’ ability to carry out their tasks effectively [28,59].

For safety concerns, an mHealth system used to guide exercise for patients with cardiac disease developed safe algorithms to detect and warn of risky situations during exercise [60]. However, the lack of an exercise companion was not mentioned in previous studies. This can be considered for mHealth, especially exercise programs that may create live animations to exercise with patients.

In this study, researchers identified the constraint of available time as a major obstacle to accessing mHealth services among patients with stroke. This encompassed challenges such as patients’ struggles with time management, inefficient use of available time, and the timely availability of caregivers [16,25,61,62]. These findings are consistent with prior studies investigating the barriers and incentives related to mHealth access among this demographic [63,64]. The effective management of time is a crucial element in the success of any mHealth intervention designed for patients with stroke [62].

The qualitative studies revealed that successful use of mHealth necessitates ongoing engagement with a caregiver, which is particularly crucial for older patients, those with more pronounced physical impairments, or those with lower digital health literacy levels. However, the effectiveness of mHealth was hampered by the limited availability of caregivers, posing challenges to sustaining quality rehabilitation approaches in home-based programs [16]. Effective time management can prove advantageous for both patients with stroke and caregivers when using telerehabilitation systems. Conversely, time constraints have been linked to decreased productivity and diminished quality within mHealth systems. Ultimately, the efficiency and efficacy of telerehabilitation hinge on the optimal use of scarce resources, notably time [65,66].

According to our findings, mHealth plays an essential role in managing and rehabilitating patients with stroke, improving and advancing the quality of care and patient outcomes, and increasing accessibility to health care resources. However, using mHealth in stroke care raises substantial concerns regarding data privacy and security. Survivors of stroke often rely on mobile applications, assisted devices for telerehabilitation, and remote monitoring tools to track their progress, communicate with health care providers, and access educational materials [24]. The data generated and transmitted through these platforms may sometimes include sensitive medical information, such as personal health records and mobility metrics. As a result, discussing mHealth in the context of stroke care must address these patient privacy and security concerns. Data transmission and storage are the most important aspects of patient privacy and security. As mobile health heavily relies on technology, cloud infrastructure, and apps, many potential risks and vulnerabilities emerge. Surprisingly, Müthing et al [67] uncovered a concerning reality within the mHealth app landscape. Their study revealed that many mHealth apps currently available lack adequate privacy and security safeguards. Even among apps certified by trusted organizations or widely adopted by the health care community, 89% were observed to transmit information digitally; alarmingly, 66% of this data was not encrypted. Moreover, there is a notable concern regarding the potential for data to be misplaced, stolen, or lost, primarily stemming from the mobile nature of the devices used in mHealth. The common habit of multiple family members sharing mobile devices like smartphones and tablets can exacerbate these concerns [68]. These risk concerns underscore the imperative requirement for solid security measures within the mHealth environment. In addition, many stakeholders have rights and responsibilities concerning an individual's medical records and the information they contain. To effectively minimize potential security risks for users in the future, it is crucial to systematically identify all parties involved and those who might be considered data “custodians” [68,69]. Informed consent, privacy policy, and access control are critical areas of concern when using mHealth. Informed consent serves as the gateway to data sharing, granting individuals or their legal representatives the authority to specify when and with whom their personal information can be shared. However, consumers often find themselves unaware of the extensive data collection and analysis methods used by mHealth services and the extent of data sharing with third parties. This issue is exacerbated by the fact that only (183/600, 30.5%) of widely used mobile health apps have privacy policies and that most apps could negatively affect users due to privacy and security violations [69]. Moreover, many mHealth industry privacy policies often resemble lengthy academic articles with language at a university level, making them daunting for the average user to navigate [68]. Therefore, to address these concerns effectively, a set of proactive steps is required, particularly when considering the needs of older adults, the majority of patients with stroke. The future focus should be on simplifying and updating existing privacy policies to make them user-friendly, age-friendly, and compliant with regulations. Collaborating and providing active support for developing a robust regulatory framework for mHealth apps are essential. This framework should empower all users, including older adults, to control their technology and decide who can access information on their mobile devices. Significantly enhancing user privacy, with specific attention to the unique requirements of older adults, is paramount. Furthermore, promoting public technology literacy and creating awareness about responsible and cautious mobile device usage through educational initiatives is vital for empowering users and ensuring the secure adoption of mHealth solutions.

Certain limitations regarding the systematic review need to be considered. First, the ages of participants in the included studies are all over 40, with most having a median age of 55 years or older. Given the paper’s focus on improving outcomes for survivors of stroke through mHealth, the older age range—whether due to sampling bias or the characteristics of patients with stroke—might negatively impact the user experience compared to younger generations. This could be attributed to varying levels of exposure to technology. Another potential limitation is the number of participants included in some of the studies. Most (7 out of 12) of the studies included fewer than 20 participants, and 10 out of 12 included fewer than 100 participants. Given the accessibility of current technology, smaller sample sizes could lead to a greater possibility of outliers, which might not adequately represent the general population of survivors of stroke, thus, introducing unintended bias. Another limiting factor is the criterion of including only English-language papers. Because stroke does not discriminate by language, including only papers written in English could omit potentially useful research regarding mHealth and survivors of stroke. Finally, this study focused solely on barriers to mHealth access in patients with stroke with a limited number of 12 included studies. To increase generalizability, future research should explore a broader range of contributing factors.

### Steps Toward the Digital Health Era

The Digital Health Era is transforming the health care paradigm through various key initiatives. One of the most significant breakthroughs in the acceptance of mHealth, telemedicine, and remote patient monitoring, which greatly improve health care accessibility by enabling remote consultations and timely interventions, particularly in remote regions, is the widespread adoption of mobile devices and advanced communication technologies [70,71]. Our study has extensively explored this aspect. The integration of wearable technology and health apps also plays a crucial role in empowering individuals to actively manage their health [72-74]. These tools monitor vital signs and activity levels and provide real-time feedback, promoting a proactive approach to wellness [75-77]. Additionally, big data analytics and AI have transformed diagnostics, treatment strategies, and predictive health care [78-80]. AI algorithms analyze vast amounts of patient data, identifying patterns, and offering valuable insights to aid health care providers in making informed decisions [78-80]. The Digital Health Era emphasizes a focus on preventive care and personalized medicine, where health care providers leverage data-driven insights to create tailored treatment plans based on an individual’s genetics, lifestyle, and specific health requirements [81,82]. Overall, the evolution toward the Digital Health Era is reshaping health care through initiatives such as telemedicine, wearable technology, big data analytics, and a focus on preventive care, personalized medicine, and user-centered design. These interventions must take into account the realities and constraints of the intended users. The advancements in different domains lay the foundation for an interlinked, data-driven health care system centered on patient requirements. Technology is vital in this evolution, enhancing patient outcomes, boosting productivity, and revolutionizing the health care experience. This ongoing amalgamation has the potential to establish a future where technology and health care converge effortlessly, leading to a more comprehensive and convenient health care environment for all.

### Conclusions

This review has highlighted significant barriers to mHealth access among patients with stroke, emphasizing the gap between mHealth’s potential and practical use. Key challenges include health-related issues, patient acceptance, infrastructure challenges, support system deficiencies, and time constraints. These findings point to the urgent need for user-friendly mHealth solutions and robust support mechanisms. Addressing these issues is critical to ensure that mHealth technologies are not only available but are also effectively adopted by survivors of stroke. Future research should focus on overcoming these barriers to enable mHealth to fully support poststroke rehabilitation and care. The goal must be to transform these obstacles into opportunities for innovation, ensuring mHealth’s role as a cornerstone of patient-centered health care.

## Appendix

Multimedia Appendix 1 Summary data.

Multimedia Appendix 2 Preferred Reporting Items for Systematic Reviews and Meta-Analyses (PRISMA) checklist.

## Abbreviations

AI: artificial intelligence
CCT: computerized cognitive training
JBI: Joanna Briggs Institute
mHealth: mobile health
MST: metacognitive strategy training
PRISMA: Preferred Reporting Items for Systematic Reviews and Meta-Analyses
VR: virtual reality
