# Supplementary material for: Current Status of Barriers to mHealth Access Among Patients With Stroke and Steps Toward the Digital Health Era: Systematic Review
Source: JMIR Mhealth Uhealth. 2024 Aug 22;12:e54511. doi: 10.2196/54511 (PMC11377914; doi:10.2196/54511)
Supplement: Multimedia Appendix 1 [file mhealth_v12i1e54511_app1.docx]

**Multimedia Appendix 1.** Summary data

| **Reference, country, year** | **Study design, Sample size, Target population, age (year)** | **The main study aim** | **Main finding****s** | **m-Health in included studies (Optional)** | **Key barriers to accessing**  **m-Health** | **Further research implication** |
| --- | --- | --- | --- | --- | --- | --- |
| (Dodakian et al., 2017)  USA  2017 | - Design: A pilot study - N = 12 - Chronic Hemiparetic Stroke - Age (Mean, SD): 54±17 | To evaluate a home-based telerehabilitation system in patients with chronic hemiparetic stroke | - Arm motor status showed significant gains, with half of the participants exceeding the minimal clinically important difference. - Although scores on computer literacy tests declined with age, neither the motor gains nor the amount of system use varied with computer literacy. - Daily stroke education via the telerehabilitation system was associated with a 39% increase in stroke prevention knowledge. - Depression scores obtained in person correlated with scores obtained via the telerehabilitation system 16 days later. - In-person blood pressure values closely matched those obtained via this system. | **A Home-Based Telerehabilitation**  **Program**   - Games focused on arm motor therapy - Therapeutic arm exercises - Stroke education for 5 minutes/day - Videoconferences 3 times/week | - Fatigue - Hardware malfunction - The conflict with other medical appointments | - This study introduced a successful home-based telerehabilitation system for chronic hemiparetic stroke patients, resulting in high compliance and significant motor gains. It also effectively integrated stroke prevention education, depression assessment, and blood pressure monitoring, potentially improving stroke rehabilitation accessibility and outcomes. - Future research could delve into the system's long-term effects, applicability across diverse stroke patient groups, and cost-effectiveness compared to traditional therapy. - Additionally, exploring broader telehealth integration beyond rehabilitation, optimizing program duration, enhancing user interfaces, and assessing psychosocial impacts and patient-centered outcomes would contribute to advancing post-stroke care through telerehabilitation. |
| (Tyagi et al., 2018)  Singapore  2018 | - Design: A qualitative study - N = 37 - Stroke (not specify), caregivers, rehabilitation therapists - Age (Mean): 59 | To explore the perceived barriers and facilitators of telerehabilitation by stroke patients, caregivers, and rehabilitation therapists in an Asian setting | The study found that telerehabilitation offers affordability and accessibility benefits, fills service gaps, and provides unexpected advantages like health monitoring. However, it also presents equipment, assessments, interfaces, and connectivity challenges. Patient-specific factors play a role in shaping rehabilitation preferences. | Not applicable | **Barriers identified by patients**   - **Equipment setup-related difficulties:** Patients encountered challenges in setting up and using the required equipment. - **Limited scope of exercises:** Patients perceived limitations in the variety and scope of rehabilitation exercises available through telerehabilitation.   **Barriers identified by tele-therapists**   - **Patient assessments:** Tele-therapists faced challenges in conducting comprehensive patient assessments remotely. - **Interface problems:** Tele-therapists encountered issues with the user interface or technology. - **Limited scope of exercises:** Similar to patients, tele-therapists noted limitations in the range of exercises they could offer. | - This study explored perceptions of telerehabilitation in Asian stroke patients, caregivers, and therapists. It found affordability and accessibility as telerehabilitation facilitators, and barriers included equipment setup, limited exercises, and connectivity issues. Patient characteristics influenced telerehabilitation preferences. - Future research directions include examining the extended benefits of telerehabilitation stroke recovery, considering cultural factors for tailored telerehabilitation interventions, developing robust technical support for older patients, exploring the feasibility and effectiveness of personalized tele-rehabilitation programs, enhancing the training of tele-therapists in virtual assessment methods, and evaluating the impact of suggested policy changes on tele-rehabilitation adoption and adherence. |
| (Dunne et al., 2020)  United Kingdom  2020 | - Design: A qualitative study - N = 66 - Stroke survivors (not specific) with partial visual loss (n = 32), caregiver (n = 10), occupational therapists (n = 24) - Age (Mean): 63.77 | To identify barriers and facilitators to using rehabilitation tools in telerehabilitation with a focus on the Durham Reading and Exploration (DREX) training application | - Findings indicate that stroke survivors' lack of confidence in technology, perceived fear of telerehabilitation, and limited face-to-face contact were major barriers to telerehabilitation. - Introducing stroke survivors to telerehabilitation requires repetition, goal setting, additional materials, and feedback because those elements can increase engagement and motivation. | Not applicable | - **Stroke survivors:** Stroke survivors believed that loss of confidence and fear of using technology prevented them from learning new things. - **Occupational therapists:** The occupational therapists reported that the limited amount of time spent with stroke survivors and their readiness to anticipate were the barriers to telerehabilitation. | Future research should consider intermediary steps, resources, multimodal approaches, training, and education prior to and during the development of telerehabilitation to increase the effectiveness and accessibility of telerehabilitation tools. |
| (Torriani-Pasin et al., 2021)  Brazil  2021 | - Design: Clinical trial (non-randomization) - N = 40 - Stroke  (26 ischemic, 11 hemorrhagic, 3 both) - Age (Mean): 55.1 | To evaluate adherence and barriers to attending a remote physical  exercise program for individuals after stroke. | The adherence rate to the program was 86.9%. The average individual attendance rate was 19.8 ± 14.8 sessions. The active participants utilized 99.1 ± 19.4 min/week of physical exercise. Participants reported one fall and 92 fears of exercising. | The remote physical exercise program included sessions for two days/week for 22 weeks, with a total of 44 sessions, which were delivered asynchronously via recorded video sessions. | - **Health-related barriers:** Lack of motor skills and physical fitness (20.6%), health appointment, constraints on exercise, lack of time, pain, behavioral issues, constrained to do mat workouts, fear of injury and fall, dual-task performance, grieve - **Environmental-related barriers**: No exercise companion, problems with communication and lack of knowledge to use Internet devices and tools, lack of a safe place to exercise, domestic life, weather - **Pandemic-related barriers**: Job commitments during a pandemic, COVID-19 issues, traveling | The research implies that remote physical exercise for stroke survivors is well accepted, but most participants prefer face-to-face programs. The benefits include increased social bonding, motivation, and self-efficacy, especially during synchronous mode. Further implication should consider the need for individualized exercises based on impairments and the severity of the impairment. |
| (Lam et al., 2022)  Hong Kong (China)  2022 | - Design: A qualitative descriptive study - N = 10 - Six strokes (not specify) and four caregivers - Age (Mean, SD): Stroke survivors 52.57±17.8, caregivers 56.75±14.86 | - To understand stroke survivors’ expectations and perceptions of a home-based virtual clinic - To understand stroke survivors’ perceptions of facilitators and barriers to using care in a virtual multidisciplinary stroke care clinic | - After a 4-week trial run of the virtual clinic service, stroke survivors and their caregivers reported a sense of security and feeling supported. - They reported having an impression of accessing the service through telecommunication technology and a willingness to learn health information. - They reported increased accessibility of rehabilitation services due to ease of use with a flexible schedule. | **A home-based virtual clinic**   - Providing a tablet containing 30 videos demonstrating appropriate self-care strategies - Communicating with a registered nurse monthly through video and telephone calls - Monitoring blood pressure regularly | - Internet connectivity - Hardware problems that hinder health monitoring (unexpected technical issues) - Interface- and design of the online platform-related issues | - This study showed a positive attitude towards telehealth services from stroke survivors and their caregivers due to ease of use, flexibility in scheduling consultation sessions, no travel needed, useful online content, and remote blood pressure monitoring. - By addressing the barriers to telehealth services, usability and acceptance can be improved to ensure successful adoption of telehealth as part of post-stroke recovery. |
| (Morse et al., 2022)  United Kingdom  2022 | - Design: Mixed method study - N = 16 - Seven stroke survivors (not specify), three partners or caregivers, and six stroke clinicians - Age (Mean, SD): Stroke survivors 67.1±6.8, partners or caregivers 51±30.1, stroke clinicians 44.7±12.1 | - To identify facilitators and barriers to the use of self-administered VR telerehabilitation - To produce recommendations that may improve future development | - All participants believed VR telerehabilitation provides more feedback than traditional therapies. - Stroke survivors reported increased engagement and enjoyment in rehabilitation. - Stroke survivors and caregivers reported psychological benefits to share negative feelings following a stroke while increasing positive feelings like confidence and independence. - Stroke survivors and caregivers reported that it is more convenient and accessible, which makes clinicians recognize the unmet need for spatial neglect rehabilitation. - The system usability scale indicated self-administered VR telerehabilitation as acceptable for all participants. - All participants were mostly confident that they could use VR for rehabilitation. - Five clinicians agreed that VR rehabilitation could improve their work performance. | **Self-administered VR telerehabilitation**   - Stroke survivors and caregivers utilize VirtualRehab, including boxing (sparring with a virtual boxing partner), bullseyes and barriers (hitting or avoiding targets), and in the kitchen (search task in realistic kitchen layout) - Stroke survivors, caregivers, and clinicians utilize Computerized spatial inattention grasping home-based therapy (c-SIGHT) - Each participant utilized the VR telerehabilitation for about 5 to 10 minutes | - Clarity of instructions - Lack of experience or confidence with technology - Equipment (expensive and limited resources like space and internet to accommodate the equipment in stroke survivors’ homes) | - Overall, stroke survivors, caregivers, and clinicians have positive attitudes towards using self-administered VR telerehabilitation regardless of their level of technology experience. - The study provides perspectives of three groups of end-users, which produce practical recommendations for future development of VR rehabilitation post-stroke. - Future research should elaborate on end-user perspectives in the early stages to improve the acceptability and engagement of interventions. The next step of this research will explore the usability and feasibility of self-administered VR rehabilitation for spatial neglect in stroke survivors’ homes. |
| (Spits et al., 2022)  Netherlands  2022 | - Design: Qualitative study - N = 11 - Stroke (not specify) - Age (Mean, SD): 66.0 ± 8.4 | - Investigated patients’ perspectives regarding the home-based training with the hoMEcare aRm rehabiLItatioN (MERLIN) system - Described the facilitators and barriers regarding upper limb training at home using MERLIN | **Body function**   - Improve shoulder function, dexterity, elbow extension, and wrist rotation. - Training cognitively challenging, improvement in their memory during the games, and feeling proud that they could do this training. - Courageous and self-confident because of the possibility of improvement in arm function.   **Personal factor**   - Acknowledged their limitations and tried to be as independent as possible. - Were motivated to improve their upper limb function.   **Environmental factors**   - Experienced mental support from their environment. - Enjoyed showing the intervention to family and friends. - Needed assistance from a caregiver.   **Training system (MERLIN)**   - In comparison to training without an assistive device, participants found MERLIN more stimulating. - The assistive device was not satisfactory for some participants because it was too big and too high, and the hand grip was too large or too small and could not be adjusted properly to the participant's hand size. - MERLIN had some malfunctions for a few participants. - Some games were not variable enough, and some participants found the cognitive component too challenging.   **Telerehabilitation platform**   - Did not mind that someone was monitoring them. - Felt a psychological strain due to the monitoring of the researcher. - It is inconvenient to use the chat function on the telerehabilitation platform to send messages back.   **Home-based training**   - Positive about the home-based telerehabilitation program, as they could control their own training schedule and divide the training into shorter sessions. - They also liked the flexibility of training whenever it was most feasible. | - MERLIN system, an assistive device and telecare platform - A robotic assistive device (ArmAssist) was used to train the upper limb function using serious games in the patient's home setting, supported by a telecare platform (Antari Homecare platform, GMV) | - Participants had to get used to the hard- and software - Some experienced psychological pressure due to the physical presence of the system in their homes - They also needed assistance from their relatives to operate the system | Future (home-based) assistive devices should be developed in a co-creation process with the different stakeholders and should take into account all the lessons we have learned so far about the implementation of new medical devices. |
| (Bhattacharjya et al., 2023)  USA  2023 | - Design: A sequential explanatory mixed-method - N = 18 - Chronic stroke (not specify) and caregivers - Age (Mean, SD): 63.19 ±13.57 | - Evaluating participants’ approach and nature of engagement with mRehab - Identifying factors that influenced the users’ engagement with mRehab. | - Extended practice may have a cumulative impact on motor performance. - The exercises were routine when using the mRehab system. - The dosage of 10 repetitions per activity, five times per week, as a barrier to using the mRehab system. - Patients who used mRehab system-compatible repetition placed themselves in high, moderate, and low groups. - Lack of time management, lack of caregiver availability, and system design problems were the barriers to using a home-based mRehab system. | **A home-based portable rehabilitation system, mReab**   - Smartphone application with 3D-printed objects - Exercise - Lab investigation | - Difficulty with time management - Lack of caregiver availability - Difficulties with the design of the system | - This study introduced a successful home-based portable telerehabilitation system for chronic non-specific stroke patients, the significance of individual tailoring home-based programs, their reception needs, and requirements. It evaluates the patients' approach and nature of engagement with the mRehab system and its outcome measures. - Evaluating Patient's Preferences in the design of home programs with technology - Future research should target intentional interviews with caregivers to understand dyadic interaction and the roles of the care recipient and caregiver as a team. It could delve into the system's long-term home program effects. - Additionally, enhancing caregiver interfaces and assessing through caregiver interviews would be important for long-term concussion. |
| (Chung et al., 2023)  South Korea  2023 | - Design: An institutional survey study - N = 156 - Patients with post-stroke paralysis (not specify) - Age (Mean, SD): 61±14 | To investigate factors affecting the decision to participate in telerehabilitation (TR) using Transcranial Direct Current Stimulation (TDCS) for motor recovery after suffering a stroke. | - 66% of patients with post-stroke paralysis participated in telerehabilitation using transcranial direct current stimulations - Earning greater than 5 million KRW (4,000 USD) was important to engaging in TR via TDCS - Preference for face-to-face and unfamiliarity of treatment where the barrier to participating in telemedicine - There is no significant difference in some socio-demographic characteristics such as sex, age, educational level, types of insurance use, stroke onset, subjective depression, motor deficit, and subjective cognitive decline for willing or unwilling to participate in Telerehabilitation therapy using TDCS. - To recover hand function, the exception of treatment effect, usefulness during a pandemic, convenience, additional personal training, and depression therapy were the common reasons to participate in TR, whereas fear of side effects, difficulty of coping, no perceived need of treatment, chronic stage of stroke, cost burdens and uncertainty regarding effect were the reason for not to participate TR. - Numerous patients who have suffered a stroke respond positively to TR using TDCS for hand function recovery. | - Telerehabilitation therapy using TDCS | - Preference for face-to-face treatment - Unfamiliarity - Fear of side effects - No perceived as treatment - Disfavoring treatment with a machine | - This study investigated factors affecting the willingness to participate in TR using TDCS for motor recovery after a stroke. - It evaluated the response of motor function of post-stroke paralysis after using telerehabilitation via TDCS, resulting in hand function being recovered. - Medical expenses were one significant barrier to participating in telerehabilitation via TDCS - Future research directions include large populations and large geographical areas, urban and rural. It includes all patients of post-stroke paralysis, mild to severe. |
| (Jaywant et al., 2023)  USA  2023 | - Design: Pilot study - N = 3 - Chronic stroke (not specific) - Age (range): 40 - 60 | - Describe the development of a remote executive function intervention combining computerized cognitive training (CCT) and metacognitive strategy training (MST). - Assess the feasibility and acceptability of this intervention in chronic stroke patients. | **High satisfaction and feasibility:** The study participants reported high satisfaction with CCT and MST. They found the intervention credible and had an expectation of change. Additionally, participants completed a high percentage of CCT and MST sessions.  **Variable clinical response**: While satisfaction and feasibility were high, the clinical response to the intervention was variable among the three participants. One participant reported a slight improvement in everyday executive functioning, while the other two demonstrated improvements in select neuropsychological tests and Complex Instrumental Activities of Daily Living (C-IADL) performance. | - Remote Computerized cognitive training (CCT) - Remote Metacognitive strategy training (MST). | - **Technology accessibility:** The study involves the use of remote interventions, which likely rely on technology. One potential barrier could be the accessibility of the required technology (e.g., smartphones, computers) for individuals with chronic stroke, especially those who may not be familiar with or have easy access to such devices. - **Technical difficulties**: The study mentions that one participant required additional phone contact with the study clinician to troubleshoot technical difficulties with the CCT software. Technical issues and challenges related to using m-Health applications or platforms could be a significant barrier. - **Digital literacy:** Participants' comfort and proficiency with technology might vary. Some individuals, especially older adults with stroke, may have limited digital literacy skills, making it challenging to use m-Health tools effectively. - **Internet connectivity:** Reliable internet access is crucial for remote interventions. Poor internet connectivity or limited access to high-speed internet in certain areas could hinder the successful implementation of m-Health interventions. - **Device compatibility:** Ensuring that m-Health applications or platforms are compatible with a range of devices (e.g., smartphones, tablets, computers) can be a barrier. Some participants may have preferred or only have access to specific types of devices. - **Privacy and security concerns**: When using m-Health applications, data privacy and security are critical considerations. Concerns about the confidentiality of personal health information may deter some individuals from participating. - **User engagement:** Maintaining participant engagement and adherence to m-Health interventions over time can be challenging. Motivating individuals to regularly use the technology and complete sessions may be a barrier. - **Training and support:** Adequate training and support for participants in using m-Health tools are essential. The study mentioned initial in-person sessions to familiarize participants with the technology, indicating that additional support may be necessary for some individuals. - **Cost of technology:** The cost associated with owning and maintaining the necessary devices and internet access can be a financial barrier for some participants, potentially limiting their participation in m-Health interventions. - **Acceptance and attitudes:** Participants' attitudes and acceptance of m-Health interventions may vary. Some individuals may be hesitant or skeptical about using technology for healthcare purposes. | **Future research direction**:  **larger randomized controlled trials:** Conduct large randomized controlled trials (RCTs) to rigorously evaluate the clinical efficacy of the combined CCT and MST intervention. To compare outcomes with and without the intervention, include control groups.  **Diverse participant groups:** Expand research to include a more diverse range of participants in terms of age, stroke severity, and comorbidities. This will help assess the intervention's effectiveness across various populations.  **Contextual factors:** Investigate how contextual factors, such as patient history, clinical presentation, and stroke characteristics (lesion size, connectivity), influence the response to intervention. This personalized approach can enhance intervention outcomes.  **Technology adaptation:** Explore the adaptability of the intervention to different technology platforms, such as web-based or app-based solutions on their phones, to make it more accessible to a wider range of stroke survivors.  **Long-term effects:** Assess the long-term effects of the intervention on participants' executive functions and daily activities. Follow-up studies can provide insights into the sustainability of gains.  **Comparative effectiveness:** Compare the effectiveness of the combined CCT and MST intervention to standalone CCT or MST interventions to determine whether the combination offers superior outcomes.  **Telehealth advancements:** Continuously adapt the intervention to advancements in telehealth services and technology, especially given the evolving landscape of remote healthcare.  **Optimal delivery mode:** Determine whether the intervention can be fully administered remotely or if occasional in-person sessions are necessary, considering practicality and effectiveness.  **Cost-benefit analysis:** Conduct cost-effectiveness analyses to assess the economic viability of implementing the intervention on a larger scale within healthcare systems. |
| (Ramaswamy et al., 2023)  USA  2023 | - Design: Mixed methods - N = 1,194 - Stroke (not specify) - Age (Mean): 62.3 | To explore possible mHealth apps to aid stroke survivors with post-stroke care and determine how demographic variables affect preferences on apps. | - People of color are more likely to find features useful (4 out of 5) - Hispanics thought finding local stroke-related resources most useful - Participants with chronic stroke (>10 years) found features less useful - Participants with no high school diploma found the most useful | **A mHealth app to aid post-stroke care**   - Ability to schedule multiple tasks - Tracking fitness and diet - Communication with others - Finding local stroke-related resources - Finding online stroke-related resources | - High cost - Complexity of use - Access to required hardware | - Future research could explore apps that could help the patient’s caretaker best care for the stroke survivors. - Additionally, exploring why less than 50% of stroke survivors in the focus group used smartphones and what may be causing that to understand better whether pursuing   m-Health is appropriate for certain demographics. |
| (Wong et al., 2023)  Hong Kong (China)  2023 | - Design: Qualitative study - N = 13 - Stroke (8 ischemic, 1 hemorrhagic) and four nurses - Age (range): 40-75 | To ascertain the experiences of stroke survivors and healthcare providers (nurses) regarding using a post-stroke telecare service. | - Telecare service is a supplementary program to complement the in-person services. - The finding stresses the idea of the desirability of integrating telecare services into mainstream - Tele-care services are effective at improving their ability to carry out their activities of daily living, which warrants its inclusion in routine services, including post-discharge support associated with sustained - Tele-care usage includes increased social support, the convenience of the tele-care service, timeliness, faster initiation of treatment, and decreased cost. | **A post-stroke telecare service**   - The goal was to use Zoom and telephone modalities by which Zoom is the main platform and telephone modalities are used as a backup. | - Technical issues and limited human resources Challenges with joining Zoom meetings and troubleshooting - Communication issues - The atmosphere associated with the face-to-face mode of follow-up was lacking - Lack of guidelines and limited training in telecare - There was a general lack of guidelines on operating a telecare service, and limited training was provided. | This qualitative research was done on using Tele-care services for stroke patients. Tele-care services are more convenient, flexible, and also affordable. There are other benefits in terms of availability and family involvement. Despite all these uses, this service is aimed to be used as an add-on to the traditional in-person checkups. It is reported that there needs to be a proper guideline for access to Tele-care services. The health care provider must also have advanced communication skills to understand their patients' verbal and nonverbal indications while using virtual assessments like Zoom. |

**Reference**

Bhattacharjya, S., Linares, I., Langan, J., Xu, W., Subryan, H., & Cavuoto, L. A. (2023). Engaging in a home-based exercise program: a mixed-methods approach to identify motivators and barriers for individuals with stroke. *Assist Technol*, 1-10. <https://doi.org/10.1080/10400435.2022.2151663>

Chung, J. E., Kim, C. T., Lee, J. E., Lee, E. J., Jung, K. I., Yoo, W. K., . . . Ohn, S. H. (2023). Factors Affecting Participation in Telerehabilitation Using Transcranial Direct Current Stimulation for Patients with Poststroke Paralysis in South Korea. *Telemedicine and E-Health*. <https://doi.org/10.1089/tmj.2022.0452>

Dodakian, L., McKenzie, A. L., Le, V., See, J., Pearson-Fuhrhop, K., Burke Quinlan, E., . . . Cramer, S. C. (2017). A Home-Based Telerehabilitation Program for Patients With Stroke. *Neurorehabilitation & Neural Repair*, *31*(10/11), 923-933. <https://doi.org/10.1177/1545968317733818>

Dunne, S., Close, H., Richards, N., Ellison, A., & Lane, A. R. (2020). Maximizing Telerehabilitation for Patients With Visual Loss After Stroke: Interview and Focus Group Study With Stroke Survivors, Carers, and Occupational Therapists. *J Med Internet Res*, *22*(10), N.PAG-N.PAG. <https://doi.org/10.2196/19604>

Jaywant, A., Mautner, L., Waldman, R., O'Dell, M. W., Gunning, F. M., & Toglia, J. (2023). Feasibility and Acceptability of a Remotely Delivered Executive Function Intervention That Combines Computerized Cognitive Training and Metacognitive Strategy Training in Chronic Stroke. *Int J Environ Res Public Health*, *20*(9). <https://doi.org/10.3390/ijerph20095714>

Lam, S. K. Y., Chau, J. P. C., Lo, S. H. S., Siow, E. K. C., Lee, V. W. Y., Shum, E. W. C., & Lau, A. Y. L. (2022). User engagement in the development of a home-based virtual multidisciplinary stroke care clinic for stroke survivors and caregivers: a qualitative descriptive study. *Disability & Rehabilitation*, *44*(20), 5983-5989. <https://doi.org/10.1080/09638288.2021.1955305>

Morse, H., Biggart, L., Pomeroy, V., & Rossit, S. (2022). Exploring perspectives from stroke survivors, carers and clinicians on virtual reality as a precursor to using telerehabilitation for spatial neglect post-stroke. *Neuropsychol Rehabil*, *32*(5), 707-731. <https://doi.org/10.1080/09602011.2020.1819827>

Ramaswamy, S., Gilles, N., Gruessner, A. C., Burton, D., Fraser, M. A., Weingast, S., . . . Levine, S. R. (2023). User-Centered Mobile Applications for Stroke Survivors (MAPPS): A Mixed-Methods Study of Patient Preferences. *Arch Phys Med Rehabil*. <https://doi.org/10.1016/j.apmr.2023.05.009>

Spits, A. H., Rozevink, S. G., Balk, G. A., Hijmans, J. M., & van der Sluis, C. K. (2022). Stroke survivors' experiences with home-based telerehabilitation using an assistive device to improve upper limb function: a qualitative study. *Disabil Rehabil Assist Technol*, 1-9. <https://doi.org/10.1080/17483107.2022.2120641>

Torriani-Pasin, C., Palma, G., Makhoul, M. P., Antonio, B. A., Lara, A. R. F., da Silva, T. A., . . . Mochizuki, L. (2021). Adherence Rate, Barriers to Attend, Safety, and Overall Experience of a Remote Physical Exercise Program During the COVID-19 Pandemic for Individuals After Stroke. *Front Psychol*, *12*, 647883. <https://doi.org/10.3389/fpsyg.2021.647883>

Tyagi, S., Lim, D. S. Y., Ho, W. H. H., Koh, Y. Q., Cai, V., Koh, G. C. H., & Legido-Quigley, H. (2018). Acceptance of Tele-Rehabilitation by Stroke Patients: Perceived Barriers and Facilitators. *Arch Phys Med Rehabil*, *99*(12), 2472-2477.e2472. <https://doi.org/10.1016/j.apmr.2018.04.033>

Wong, A. K. C., Bayuo, J., Wong, F. K. Y., Kwok, V. W. Y., Tong, D. W. K., Kwong, M. K., . . . Li, W. C. (2023). Sustaining telecare consultations in nurse-led clinics: Perceptions of stroke patients and advanced practice nurses: A qualitative study. *Digit Health*, *9*, 20552076231176163. <https://doi.org/10.1177/20552076231176163>
